# Checkpoint for Considering Interleukin-6 as a Potential Target to Mitigate Secondary Brain Injury after Cardiac Arrest

**DOI:** 10.3390/brainsci14080779

**Published:** 2024-07-31

**Authors:** Jung A Yoon, Yeonho You, Jung Soo Park, Jin Hong Min, Wonjoon Jeong, Hong Joon Ahn, So Young Jeon, Dongha Kim, Changshin Kang

**Affiliations:** 1 Department of Emergency Medicine, Chungnam National University Hospital, 282 Munhwa-ro, Jung-gu, Daejeon 35015, Republic of Korea; liolio1108@cnuh.co.kr (J.A.Y.); yyo1003@naver.com (Y.Y.); cpcr@cnu.ac.kr (J.S.P.); gardenjun@hanmail.net (W.J.); jooniahn@daum.net (H.J.A.); chloe9899@cnuh.co.kr (S.Y.J.); 2 Department of Emergency Medicine, College of Medicine, Chungnam National University, 282 Mokdong-ro, Jung-gu, Daejeon 35015, Republic of Korea; laphir2006@naver.com; 3 Department of Emergency Medicine, Chungnam National University Sejong Hospital, 20, Bodeum 7-ro, Sejong 30099, Republic of Korea; 4 Department of Statistics, Sungshin Women’s University, 2, Bomun-ro, Seongbuk-gu, Seoul 02844, Republic of Korea; dongha0718@sungshin.ac.kr

**Keywords:** heart arrest, interleukin 6, post-cardiac arrest syndrome, injury severity scores

## Abstract

Interleukin-6 (IL-6) was suggested as a potential target for intervention to mitigate brain injury. However, its neuro-protective effect in post-resuscitation care has not been proven. We investigated the time-course of changes in IL-6 and its association with other markers (systemic inflammation and myocardial and neuronal injury), according to the injury severity of the cardiac arrest. This retrospective study analyzed IL-6 and other markers at baseline and 24, 48, and 72 h after the return of spontaneous circulation. The primary outcome was the association of IL-6 with injury severity as assessed using the revised Post-Cardiac Arrest Syndrome for Therapeutic Hypothermia scoring system (low, moderate, and high severity). Of 111 patients, 22 (19.8%), 61 (55.0%), and 28 (25.2%) had low-, moderate-, and high-severity scores, respectively. IL-6 levels were significantly lower in the low-severity group than in the moderate- and high-severity groups at baseline and at 24 h and 72 h (*p* < 0.005). While IL-6 was not independently associated with neuronal injury markers in the low-severity group, it was demonstrated to be associated with it in the moderate-severity (β [95% CI] = 4.3 [0.1–8.6], R^2^ = 0.11) and high-severity (β [95% CI] = 7.9 [3.4–12.5], R^2^ = 0.14) groups. IL-6 exhibits distinct patterns across severity and shows differential associations with systemic inflammation or neuronal injury.

## 1. Introduction

During cardiac arrest, patients experience whole-body ischemia, which is followed by reperfusion injuries after the return of spontaneous circulation (ROSC). Both ischemia and reperfusion injuries activate inflammatory cascades, leading to a systemic inflammatory response or sepsis-like syndrome [1,2,3].

Among the various inflammatory markers, interleukin-6 (IL-6) has been identified as a reliable and predictive marker of outcome [4,5], and it is a potential target for intervention to mitigate the secondary brain injury strongly associated with the outcome [6,7]. The available data suggest that IL-6 is a potential target to mitigate secondary brain injury after cardiac arrest. A clinical trial (known as the IL-6 Inhibition for Modulating Inflammation After Cardiac Arrest (IMICA) randomized clinical trial) for the effect of an IL-6 receptor antibody (tocilizumab) with the aim of limiting the inflammatory response after out-of-hospital cardiac arrest (OHCA) was published recently [6,7]. Although systemic inflammation and myocardial injuries were significantly reduced, intervention with tocilizumab did not show any neuroprotective effects [6,7].

However, the IMICA trial was limited because it was performed with a limited phenotype of cardiac arrest. Therefore, we hypothesized that IL-6 levels are heterogeneously associated with the severity of the cardiac arrest injury. Thus, this study aimed to investigate the association of IL-6 with the severity of cardiac arrest and the independent relationship between IL-6 and other biomarkers of systemic inflammation and myocardial or neuronal injury for each severity level of cardiac arrest.

## 2. Materials and Methods

### 2.1. Study Design and Population

This was a single-center, retrospective, observational, registry-based study. This registry in a tertiary-care hospital (Chungnam National University Hospital in Daejeon, Republic of Korea; CNUH) prospectively collected data from patients receiving post-cardiac arrest care after OHCAs between October 2018 and January 2023. The institutional review board approved the study protocol before data collection began (CNUH-2023-03-092). Adult patients (aged > 18 years) who were undergoing post-cardiac arrest care after OHCAs were included in this study. Patients who underwent extracorporeal membrane oxygenation, had died due to cardiac death within 24 h after the ROSC, or had undetermined injury severity were excluded from this study.

### 2.2. Post-Cardiac Arrest Care

Patients with a Glasgow Coma Scale (GCS) score of less than 6 after the ROSC underwent post-cardiac arrest care. Targeted temperature management (TTM) was performed using an external cooling device (Arctic Sun^®^ 5000, BD, Franklin Lakes, NJ, USA). The targeted temperatures of 33 or 36 °C were maintained for 24 h, with rewarming to 37 °C at a rate of 0.25 °C per hour, and this was monitored with an esophageal or bladder temperature probe. Sedatives (midazolam) and paralytics (cisatracurium and rocuronium) were administered. If there was evidence of electrographic seizure or a clinical diagnosis of seizure, antiepileptic drugs such as lorazepam, levetiracetam, and valproate were administered. All patients received standard intensive care according to our institutional intensive care unit protocol based on the 2021 International Guidelines for Post-Cardiac Arrest care [8].

Since 2018, the withdrawal of life-sustaining therapy (WLST) has been highly restrictively authorized in Korea, with rigorous qualification criteria for brain death, such as a flat (<2 µV) in an electroencephalogram for 30 min [9,10]. Legally, qualifying for brain death must require declarations for an irreversible and unrecoverable status from at least two physicians, even if the family is willing to consent to this status. Therefore, the physicians in charge of post-cardiac arrest care not only did not encourage the WLST in our patients, but it was also restricted to patients with brain death who were denied organ donation from caregivers or families.

### 2.3. Data Acquisition

#### 2.3.1. Baseline Characteristics

The following variables were extracted from the data registry: age, sex, Charlson comorbidity index, sequential organ failure assessment (SOFA) score during post-cardiac arrest care, witnessed collapse, bystander cardiopulmonary resuscitation (CPR), time from CPR to the ROSC (anoxic time), first monitored rhythm, etiology of cardiac arrest, Glasgow Coma Scale score after the ROSC, and time to measure the baseline biomarkers. Neurological outcomes were assessed six months after the OHCA using patients’ Cerebral Performance Category (CPC) scores, which classify patients into the following five categories: CPC 1 (good performance), CPC 2 (moderate disability), CPC 3 (severe disability), CPC 4 (vegetative state), and CPC 5 (brain death or death). Good neurological outcomes were defined as CPC scores of 1 or 2.

#### 2.3.2. Measurement of IL-6, Systemic Inflammatory, Myocardial Injury, and Neuronal Injury Markers

Blood samples were collected immediately after applying the TTM device and at 24, 48, and 72 h after the ROSC. An analysis of IL-6 in the serum samples was performed using ELISA, according to the manufacturer’s recommendations (Roche Diagnostics GmbH, Mannheim, Germany), with a detection limit of 1.5 pg/mL. As neuronal injury markers, NSE levels were measured using an electrochemiluminescence immunoassay (ECLIA) with Elecsys NSE^®^ (COBAS e801; Roche Diagnostics, Rotkreuz, Switzerland) and extracted from our prospectively collected cardiac arrest registry. The measurement range for NSE was 0.1–300 ng/mL. C-reactive protein (CRP) and procalcitonin (PCT) were used as systemic inflammatory markers. CRP levels were measured using an immunoturbidimetric assay (CRPL3; Roche Diagnostics, Indianapolis, IN, USA), and the reference range was 0.0–30.0 mg/dL. PCT was measured using an Elecsys BRAHMS procalcitonin automated electrochemiluminescence assay (BRAHMS, Henningsdorf, Germany) on a Roche Cobas e-System (Roche Diagnostics, Basel, Switzerland) with a reference range of 0.05–200.0 ng/mL. Creatine kinase-MB (CK-MB) and cardiac troponin I (TnI) levels were also extracted from our registry as myocardial injury markers. The CK-MB levels were measured using an electrochemiluminescence immunoassay with a Modular Analytics E170 immunology analyzer (Roche Diagnostics, Mannheim, Germany), and the reference range was 0.1–300.0 ng/mL. The levels of TnI were measured by a chemiluminescence immunoassay using an ADVIA Centaur instrument (Siemens, Munich, Bavaria, Germany), and the reference range was 1–50,000 pg/mL.

#### 2.3.3. Assessment for Injury Severity of the Cardiac Arrest

The cohort was divided into three groups based on the severity of the ischemic period using the revised Post-Cardiac Arrest Syndrome for Therapeutic Hypothermia (rCAST) scoring system, which was developed to determine the severity of individual injuries during the ischemic period. It was internally and externally validated in previous studies as a reliable scoring system to assess injury severity in the ischemic period in patients with OHCAs [11,12,13]. The rCAST score was calculated immediately after the ROSC or upon arrival at the hospital. Five clinical parameters (initial rhythm, witnessed arrest/low-flow time (CPR to the ROSC), blood pH, serum lactate, and the motor score of GCS at the time of the ROSC) were used to calculate the rCAST score, and then the patients with an ROSC after OHCA were classified into the following risk categories: low-severity group, with an rCAST score of less than or equal to 5.5; moderate-severity group, with an rCAST score of less than 6 and greater than or equal to 14.5; and high-severity group, with an rCAST score of greater than 14 [11].

### 2.4. Assessment for the Injury Severity of the Cardiac Arrest

The primary outcome was the time-course of the changes in the IL-6 levels over time for each injury severity, and this was estimated using the rCAST degree (low, moderate, or high) and its independent association with other biomarkers (NSE, CRP, PCT, CK-MB, and TnI).

### 2.5. Statistical Analysis

Categorical and continuous variables were presented as counts with percentiles and median values with interquartile ranges (IQR), respectively. The categorical variables were compared between the groups using a chi-square test or Fisher’s exact test, as appropriate. The continuous variables were compared between the groups using a Mann–Whitney U-test. The data for the biomarkers were longitudinal, and the observations were cross-sectional over several periods. For the statistical analyses, we used a generalized linear mixed model (GLMM) with a random intercept to examine whether the longitudinal changes in the biomarkers over 72 h differed by group while accounting for the within-person correlations in the outcomes, as our dataset had a non-normal distribution [14]. To test our primary hypotheses, we included injury severity, time, and the interaction terms between the group and time in the GLMM. The homoscedasticity and multi-collinearity checks were performed under the model adequacy test requirements of acceptance of the fitted models. Multiple linear and logistic regression analyses were performed for each group of patients. A multivariate linear regression analysis determined the relationship between the IL-6 levels and the other biomarkers. Backward selection was used to develop the final adjusted model. The logistic regression analysis results were expressed as parameter estimates with 95% confidence intervals (CI). The statistical analyses were performed using IBM IBM-SPSS 26.0 for Windows (IBM Corp., Armonk, NY, USA) and Prism 10.0 (GraphPad, San Diego, CA, USA). The significance level was set at *p* < 0.05.

## 3. Results

### 3.1. Baseline Characteristics of the Cohort

**Figure 1:**
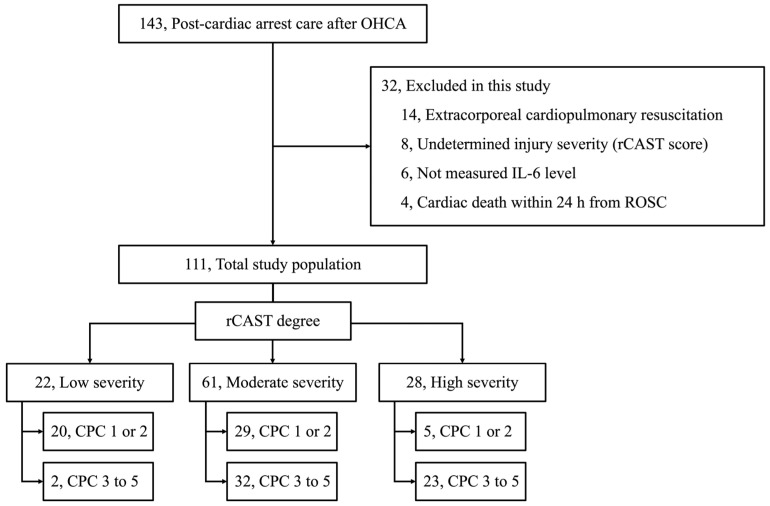
Flow diagram of the included study population enrollment. Abbreviations: CPC, cerebral performance category; OHCA, out-of-hospital cardiac arrest; rCAST, revised Post-Cardiac Arrest Syndrome for Therapeutic hypothermia; ROSC, return of spontaneous circulation.

Of the 143 patients who underwent post-resuscitation care after OHCAs, 32 were excluded (14 achieved exclusion due to extracorporeal CPR, 8 due to undetermined injury severity due to insufficient information from a prior medical facility, 6 due to missing IL-6 measurements, and 4 due to cardiac death within 24 h after the ROSC). Finally, 111 patients were enrolled in this study (Figure 1) and divided into 3 groups according to the degree of rCAST. Twenty-two (19.8%), sixty-one (55.0%), and twenty-eight (25.2%) patients belonged to the low-, moderate-, and high-severity groups, respectively (Figure 1). Several cardiac arrest characteristics, such as witnessed arrest, bystander CPR, shockable rhythm, cardiac etiology, anoxic time, and GCS after the ROSC, were significantly different according to the injury severity of the cardiac arrest (i.e., low- vs. moderate- vs. high-severity groups; Table 1). Good neurological outcomes and survival to discharge were observed in 20 (90.9%)/20 (90.9%), 30 (47.5%)/36 (59.0%), and 5 (17.9%)/8 (28.6%) patients in the low-, moderate-, and high-severity groups, respectively (Figure 1 and Table 1). 

### 3.2. Time-Course of the Changes in IL-6 Associated with Injury Severity

**Figure 2:**
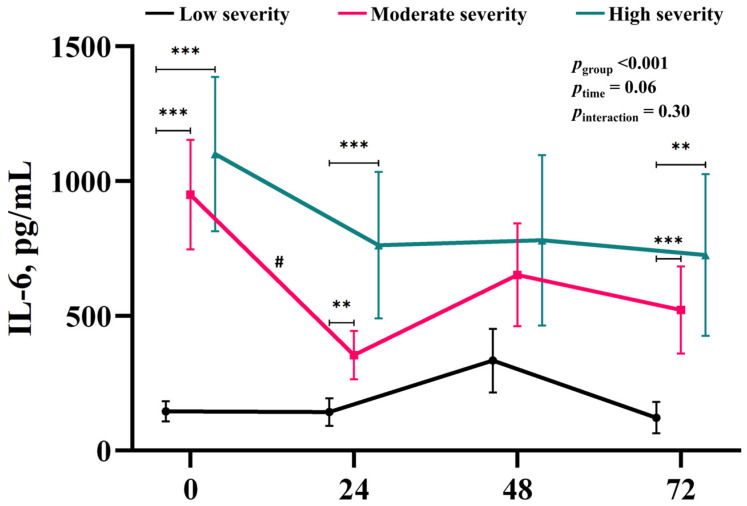
Time-course of the changes in the IL-6 levels over time (*x*-axis; 0, 24, 48, and 72 h from the ROSC) in each severity group (low-severity, black; moderate-severity, red; and high-severity, green). A statistical analysis was performed using generalized estimating equation methods, and thus, the biomarker levels and their variabilities are presented as mean values and standard errors. The adjusted *p*-values are presented, followed by the post hoc test results, where ** indicates *p*_group_ < 0.01, and *** indicates *p*_group_ < 0.001 for the inter-group analysis and # denotes *p*_time_ < 0.05 for the longitudinal analysis within the groups. Abbreviations: IL-6, interleukin-6; ROSC, return of spontaneous circulation.

While no significant effect of time (*p*_time_ = 0.06) or significant interaction between injury severity and time (*p*_interaction_ = 0.30) were noted, significant differences between the groups were observed in the post hoc test (Figure 2). IL-6 levels in the moderate- and high-severity groups were significantly higher than those in the low-severity group at baseline (low vs. moderate and high severity, 145.2 pg/mL ± 46.1 vs. 949.7 pg/mL ± 182.6 and 1100.7 pg/mL ± 309.9, *p* < 0.001 and *p* = 0.002, respectively), 24 h (low vs. moderate and high severity, 143.3 pg/mL ± 45.5 vs. 401.1 pg/mL ± 76.5 and 762.3 pg/mL ± 214.6, *p* = 0.004 and *p* = 0.005, respectively), and 72 h (low vs. moderate and high severity, 122.4 pg/mL ± 40.5 vs. 521.5 pg/mL ± 105.7 and 725.1 pg/mL ± 207.9, *p* < 0.001 and *p* = 0.004, respectively; Figure 2 and Table 2). In a post hoc test for the time difference, only the moderate-severity group showed a decrease in IL-6 for the first 24 h (baseline vs. 24 h, 949.7 ± 182.6 vs. 401.1 ± 76.5, *p* = 0.006, Figure 2 and Table 2).

### 3.3. Time-Course of the Changes in the Other Biomarkers Associated with Injury Severity

Table 2 shows the time-course of the changes in the levels of the markers for neuronal injury (NSE), systemic inflammation (CRP and PCT), and myocardial injury (CK-MB and TnI). NSE levels were significantly higher in the moderate- and high-severity groups than they were in the low-severity group for all time points, whereas there were no significant differences between the moderate- and high-severity groups. NSE levels in the moderate- and high-severity groups showed an increasing pattern over time, whereas there was no trend towards an increase in the low-severity group. While PCT levels were significantly higher in the moderate- and high-severity groups than those in the low-severity group for all time points (peaking at 24 h), the CRP changes exhibited a different pattern. Neither the CK-MB nor the TnI levels showed clear differences between the groups. In particular, TnI levels were not significantly different between the groups over time. They presented a similar pattern over time, peaking at 24 h and then decreasing.

**Table 2:** Time-course changes in the biomarker levels according to injury severity.

Biomarker	Time	Injury Severity			*p*_group_ ^a^	*p*_time_ ^a^
		Low, 22	Moderate, 61	High, 28		
NSE, ng/mL	baseline	24.4 ± 1.4, 22	48.7 ± 6.7, 61 *	63.2 ± 13.7, 28 *	<0.001	0.05
	24 h	28.1 ± 3.2, 21	68.7 ± 9.7, 60 *	96.9 ± 18.4, 28 *		
	48 h	24.7 ± 3.1, 21	76.1 ± 11.8, 59 *	114.0 ± 19.7, 27 *		
	72 h	27.4 ± 6.4, 21	68.5 ± 13.8, 58 *	121.9 ± 18.2, 26 *		
CRP, mg/dL	baseline	1.5 ± 0.7, 21	1.2 ± 0.3, 59	1.4 ± 0.6, 26	0.18	<0.001
	24 h	2.8 ± 0.5, 21	4.1 ± 0.5, 57 *	4.7 ± 0.9, 26 *		
	48 h	5.2 ± 0.7, 19	8.5 ± 0.8, 55 *	8.0 ± 1.1, 23		
	72 h	5.5 ± 1.0, 19	8.2 ± 0.8, 54	8.4 ± 1.2, 24		
PCT, ng/mL	baseline	0.4 ± 0.1, 12	2.0 ± 0.6, 34 *	3.3 ± 1.2, 22 *	<0.001	0.01
	24 h	0.5 ± 0.1, 20	12.9 ± 4.7, 46 *	20.8 ± 8.0, 24 *		
	48 h	0.4 ± 0.1, 20	8.8 ± 2.1, 46 *	15.2 ± 6.3, 26 *		
	72 h	0.5 ± 0.1, 13	7.4 ± 2.1, 37 *	12.5 ± 5.5, 22 *		
CK-MB, ng/mL	baseline	11.5 ± 3.3, 22	20.7 ± 4.4, 60	11.2 ± 2.0, 28	0.006	<0.001
	24 h	48.7 ± 17.2, 19	82.2 ± 14.1, 40	66.5 ± 12.3, 24		
	48 h	31.1 ± 16.5, 18	61.6 ± 12.4, 41 *	60.2 ± 16.2, 21 *		
	72 h	9.6 ± 3.7, 18	15.6 ± 3.1, 40	20.9 ± 9.2, 21 *		
TnI, pg/mL	baseline	613.7 ± 410.9, 22	1310.2 ± 473.7, 60	330.7 ± 68.1, 28	0.06	0.97
	24 h	2528.9 ± 1355.3, 18	7557.2 ± 1668.8, 37	2811.0 ± 737.4, 22		
	48 h	2007.9 ± 1534.7, 17	4890.9 ± 1247.9, 39	2022.5 ± 780.1, 21		
	72 h	1169.8 ± 895.3, 18	3745.4 ± 1136.0, 39	2045.5 ± 1008.6, 21		

Biomarker levels and their variability are presented as mean values and standard errors due to the statistical analysis using GLMM. The number of measurements was determined immediately after the means and standard errors for each case. No significant differences between the moderate- and high-severity groups were observed for any of the biomarkers or time points. ^a^, *p*-values were adjusted with other biomarker levels using the GLMM statistical analysis. *, significant difference (*p* < 0.05) for the low-severity group according to the GLMM analysis, with a Bonferroni post hoc test. Abbreviations: NSE, neuron-specific enolase; CRP, C-reactive protein; PCT, procalcitonin; CK-MB, creatinine kinase-myocardial band; TnI, troponin I; GLMM, generalized linear mixed model.

### 3.4. Independently Associated Biomarkers for IL-6 Level

Analysis of the independent association of IL-6 with the other biomarkers according to the injury severity revealed that IL-6 was not associated with any other biomarker in the low-severity group, whereas IL-6 in the moderate- and high-severity groups was independently associated with PCT (β [95% CI] = 17.0 [5.9 to 28.2], *p* = 0.003, R^2^ = 0.11) and NSE (in the moderate-severity group, β [95% CI] = 4.3 [0.1 to 8.6], *p* = 0.04, adjusted R^2^ = 0.11, and in the high severity group, β [95% CI] = 7.9 [3.4 to 12.5], *p* = 0.001, adjusted R^2^ = 0.14), respectively (Table 3).

## 4. Discussion

This study included most phenotypes of patients who received post-resuscitation care after the ROSC in a non-WLST setting. Consequently, this study demonstrated the time-course of changes in IL-6 levels across the overall spectrum of cardiac arrest phenotypes. As anticipated, the time-course of IL-6 levels exhibited distinctions by the injury severity of the cardiac arrest, as estimated using the following well-validated scoring system: the rCAST grading system (low vs. moderate vs. high severity). In addition, the independent association of IL-6 with other biomarkers revealed variability in the severity of the cardiac arrest.

The IMICA trial demonstrated the beneficial effect of tocilizumab, a humanized IL-6 receptor monoclonal antibody, in reducing the biomarker levels of systemic inflammation and myocardial injury [7]. In addition, secondary analyses have revealed the potential role of anti-inflammatory agents in secondary systemic injuries after OHCAs [15]. However, one disappointing point is that the primary determinant of outcome after cardiac arrest is the presence of a hypoxic ischaemic brain injury (HIBI), with an emphasis on the brain injury following cardiac arrest [2]. Although the primary injury causes substantial neuronal loss, post-resuscitation additive cerebral injuries (i.e., secondary brain injuries) account for significant cerebral ischemia and cellular death [2,16]. Therefore, the management of HIBI after cardiac arrest has focused on preventing or minimizing secondary brain injuries [16]. Additional pharmacological agents might have the potential to produce breakthrough effects on the cascade of secondary brain injuries following cardiac arrest. Therefore, it is highly likely that therapies targeting IL-6 or other aspects of inflammation will require brain-targeting to yield measurable improvements in neurological outcomes [15]. However, the IMICA trial did not demonstrate any potential benefits in patients with brain injuries. Here, we discuss some checkpoints for developing future clinical trials of advanced pharmacological therapies targeting secondary brain injuries following cardiac arrest based on the limitations of the IMICA trial and our findings. 

We demonstrated that the low-severity group exhibited significantly lower levels of IL-6 at baseline and 24 h than the moderate- and high-severity groups. Additionally, there were no increases in IL-6 levels during the first 24 h in the low-severity group under generalized post-resuscitation care, which did not involve any additional intervention. These findings suggest that the anti-inflammatory effect of tocilizumab may be underestimated in patients with low-severity cardiac arrest compared to those with moderate- or high-severity cardiac arrest. It is well-established in medicine, particularly in critical care, that the effect of a treatment depends on the severity of the injury or disease. For instance, temperature control (hypothermia vs. normothermia), the standard-of-care post-resuscitation, has shown different results based on the severity of the cardiac arrest injury in large trials (shockable vs. non-shockable) [17,18]. The early injury severity of cardiac arrest, as measured by the rCAST scoring system, which was well-validated in a previous study [11], has also been reported to be significantly associated with survival, good outcomes, and cause of death [19]. 

Furthermore, the moderate severity estimated using the rCAST scoring system may independently offer a neuroprotective advantage over the presence of hypothermia [20]. Therefore, several studies have suggested that the severity of cardiac arrest influences overall outcomes in patients undergoing post-resuscitation care. In the IMICA study, notably, only one patient among the survivors had a poor neurological outcome (CPC 3–5). Additionally, the median values of the NSE levels measured 48 and 72 h after cardiac arrest were near normal (17–18 ng/mL), indicating a good neurological outcome with reasonable prognostic accuracy [21]. This suggests that the IMICA trial predominantly enrolled patients with mild brain injuries, which likely corresponded to the low-severity ranking of the rCAST grading system. Our finding that IL-6 was independently associated with NSE, a marker of neuronal injury, only in cases beyond mild brain injury (i.e., moderate to high severity) supports this potential limitation of the IMICA study. Given this limitation and the lack of an association between IL-6 and NSE in patients with low-severity cardiac arrest, the brain inflammatory response that can exacerbate HIBI after cardiac arrest is insufficient for determining the neuroprotective effect of tocilizumab. Therefore, we suggest that the narrow selection bias raises questions regarding the generalizability of the neuroprotective effect of tocilizumab against IL-6 because their limited cohort did not represent the full spectrum of cardiac arrest severity. This selection bias may have led to an underestimation of the effect of tocilizumab, as the patients with relatively low IL-6 levels may not have exhibited the full therapeutic potential of the intervention.

In this study, the NSE levels showed consistent pattern towards increases, whereas the CRP and myocardial injury markers (CK-MB and TnI) showed fluctuating levels with distinct peaks. These results are generally in line with those from previous studies [6,7]. However, PCT was newly included in this analysis to investigate its association with IL-6. The PCT levels peaked at 24 h and then decreased over time, with higher levels observed in the high-severity group compared to the low-severity group, possibly due to the relatively higher infection rate in the former. The markers for myocardial injury followed a typical pattern, in line with what has already been described in the field. However, no specific association was found between IL-6 and these markers. In the multivariable linear regression analysis, we showed that IL-6 was associated with NSE and PCT levels in the moderate- and high-severity groups, whereas the markers for myocardial injury did not show an association with IL-6. In line with our findings, several previous studies have shown that NSE is strongly associated with poor neurological outcome [8,16], and PCT seems to account for injury severity associated with cardiac arrest and its outcome rather than infection levels [22]. In particular, our cohort predominantly included cases of confirmed permanent brain injury (56, 50.5%) or neuronal death (30, 63.8%; among non-survivors), even after 72 h of post-resuscitation care based on the very limited WLST policy in Korea. We suggest that the characteristics of the study population may have influenced the association of IL-6 with the other biomarkers. 

As a major concern for the neuroprotective effect of tocilizumab in patients with cardiac arrest, the authors suggested that there is no conclusive evidence on whether tocilizumab crosses the blood–brain barrier (BBB) in humans, whether healthy or ill [23]. The BBB is a selective barrier that regulates the entry of substances from the bloodstream into the central nervous system [23]. Neuroprotective drugs must cross the BBB at sufficient concentrations to be effective. Increasing BBB permeability may improve the delivery of neuroprotective agents to the brain, improving the efficacy of a drug [24]. A recently published study showed that the phenotypes of patients who underwent post-resuscitation care after mild-injury-severity cardiac arrest—likely corresponding to the patients enrolled in the IMICA trial—not only showed non-significant time-courses of changes in BBB status but also that their median BBB permeability did not reach severe BBB disruption [25]. In addition, an experimental study using a healthy, nonhuman primate model demonstrated that intravenously administered tocilizumab has very poor cerebrospinal fluid penetration [23]. Hence, we suggest that the limited phenotypes of the enrolled patients in the IMICA trial (i.e., those with a likely nearly normal BBB status due to the mild injury severity of the cardiac arrest) may lead to the underestimation of the neuroprotective effect of tocilizumab. Confirmation of BBB permeability could be used to deliver novel pharmacological treatments to the brain where an otherwise intact BBB would prevent them from reaching their target [26,27]. Barton and Elmer suggested that BBB-targeted early-phase therapeutic trials are warranted to answer a long-standing question: is the alteration of BBB permeability an innocent bystander or is it the causal pathway of secondary brain injury [28]? We suggest that a study on the neuroprotective effects of tocilizumab or other pharmacological agents in patients receiving post-resuscitation care will be an important step in our field. However, it is essential to establish the proper enrolment of patients, taking into account the severity of their neurological injury/disease, including BBB status.

This study had some limitations. First, this was a retrospective study performed at a single center. Due to the characteristics of this study, bias from the variability in the number of measurements according to the sampling time and type of biomarker could not be avoided. Second, high standard deviations were observed in the IL-6 level values. This substantial variability within the groups indicated a large amount of heterogeneity among the participants, which could have complicated the detection of statistically significant differences between the groups. The high variability reduced the precision of our estimates and may have impacted the robustness of our findings. In addition, the multivariable analysis presented low adjusted R^2^ values, strongly suggesting that a large part of the variability in the IL-6 levels remained unexplained. To address both of these limitations, we suggest that future studies should consider larger sample sizes to increase the power of the analysis, with adjustment for the co-variables related to the inflammation cascade. Third, potential cases of self-fulfilling prophecy in terms of prognosis prediction could not be excluded because the results of the IL-6 levels were available to the physicians participating in the study. Fourth, the maximum measurement range value of IL-6 was 5000 pg/mL, and IL-6 levels above the measurement range were not diluted or analyzed. Fifth, there are many unknown aspects of IL-6 as an inflammatory cytokine, and further studies on the pathophysiology of IL-6 are required. Sixth, other cytokines and inflammatory markers (e.g., erythrocyte sedimentation rate) were not measured. Seventh, the 24 h interval for the biomarker measurement presented a significant limitation for the real time-course analysis. This sampling frequency may have been insufficient for capturing rapid fluctuations in the biomarker levels that may occur within shorter periods of time. Therefore, this low resolution may have led to an incomplete and potentially misleading representation of the dynamic profile of a particular biomarker. However, several studies on biomarkers in post-resuscitation care have adopted the 24 h interval [8,16,21,22]. We suggest that this approach should be used to balance the need for practical and consistent measurement intervals with the goal of minimizing the impact of missing short-term fluctuations. Thus, we could not speculate about the changes in these parameters. Further multi-center studies are required to confirm the generalizability of these results.

## 5. Conclusions

The time-course of changes in IL-6 levels during post-resuscitation care has an individual pattern when stratified by injury severity. Significantly lower IL-6 levels manifest in patients with low-severity cardiac arrest than in those with moderate- or high-severity cardiac arrest. Serum IL-6 levels are differentially associated with systemic inflammation or neuronal injury, depending on the severity of the cardiac arrest. An appropriate cardiac arrest phenotype should be considered in future clinical trials of advanced therapies for neuroprotective effects after cardiac arrest. However, further studies are required to confirm these findings.

## Figures and Tables

**Table 1:** Baseline demographics and characteristics.

Variables	Total Cohort, 111	Injury Severity			*p*
		Low, 22	Moderate, 61	High, 28	
Age, years	58 (41–68)	60 (46–71)	60 (49–68)	50 (35–62)	0.06
Sex, male	85 (76.6)	19 (86.4)	44 (72.1)	22 (78.6)	0.39
Comorbidities					
Myocardial infarction	15 (13.5)	3 (13.6)	10 (16.4)	2 (7.1)	0.50
Peripheral vascular disease	4 (3.6)	1 (4.5)	2 (3.3)	1 (3.6)	0.96
Congestive heart failure	9 (8.1)	2 (9.1)	6 (9.8)	1 (3.6)	0.59
COPD	5 (4.5)	1 (4.5)	1 (1.6)	3 (10.7)	0.16
Chronic kidney disease	16 (14.4)	5 (22.7)	8 (13.1)	3 (10.7)	0.44
Previous cardiac arrest	0 (0)	0 (0)	0 (0)	0 (0)	
Charlson comorbidity index	2 (1–4)	2 (1–6)	3 (1–5)	1 (0–3)	0.22
Cardiac arrest characteristics					
Witnessed	73 (65.8)	22 (100.0)	40 (65.6) **	11 (39.3) ***	<0.001
Bystander CPR	79 (71.2)	20 (90.9)	37 (60.7) *	22 (78.6)	0.02
Shockable rhythm	42 (37.8)	14 (63.6)	22 (36.1)	6 (21.4) *	0.009
Cardiac etiology	55 (49.5)	16 (72.7)	31 (50.8)	8 (28.6) *	0.008
Anoxic time ^a^, min	20 (9–30)	1 (0–1)	1 (0–12)	5 (1–24) **	0.003
GCS score after the ROSC	3 (3–4)	4 (3–6)	3 (3–4) **	3 (3–3) ***	<0.001
SOFA score at admission	10 (7–12)	8 (7–11)	10 (8–12)	11 (9–13)	0.67
Cause of cardiac arrest					
Cardiogenic	54 (48.6)	17 (77.3)	29 (47.5)	8 (28.6) **	0.02
Hypoxia	43 (38.7)	3 (13.6)	26 (42.6)	14 (50.0) **	
Nephrogenic	5 (4.5)	1 (4.5)	3 (4.9)	1 (3.6) **	
Others	9 (8.1)	1 (4.5)	3 (4.9)	5 (17.9) **	
Echocardiography after the ROSC					
Left ventricle contractility					
Normal	59 (53.2)	9 (40.9)	33 (54.1)	17 (60.7)	0.43
Mild dysfunction	7 (6.3)	3 (13.6)	4 (6.6)	0 (0)	
Moderate dysfunction	33 (29.7)	8 (36.4)	16 (26.2)	9 (32.1)	
Severe dysfunction	12 (10.8)	2 (9.1)	8 (13.1)	2 (7.1)	
Right ventricle dysfunction	5 (4.5)	1 (4.5)	3 (4.9)	1 (3.6)	0.96
Regional wall motion abnormality					
LAD territory	12 (10.8)	6 (27.3)	3 (4.9)	3 (10.7)	0.05
LCx territory	6 (5.4)	2 (9.1)	4 (6.6)	0 (0)	
RCA territory	2 (1.8)	0 (0)	2 (3.3)	0 (0)	
CAG performed ^b^					
1VD	5 (4.5)	2 (9.1)	2 (3.3)	1 (3.6)	0.52
2VD	3 (2.7)	1 (4.5)	2 (3.3)	0 (0)	
3VD	3 (2.7)	0 (0.0)	3 (4.9)	0 (0)	
PCI performed before TTM ^c^	7 (6.3)	2 (9.1)	4 (6.6)	1 (3.6)	0.72
Presented infection					
Pulmonology	7 (6.3)	1 (4.5)	4 (6.6)	2 (7.1)	0.93
Genitourinary	1 (0.9)	0 (0)	1 (1.6)	0 (0)	
Neck	1 (0.9)	0 (0)	1 (1.6)	0 (0)	
Administration of steroid, mg/kg	160 (61–536)	91 (31–371) ^d^	60 (11–381) ^e^	240 (80–720) ^f^	0.11
Outcome					
Good neurological outcome	54 (48.6)	20 (90.9)	29 (47.5) ***	5 (17.9) ***^, †^	<0.001
Survival at discharge	64 (57.7)	20 (90.9)	36 (59.0) *	8 (28.6) ***^, †^	<0.001

Data are presented as *n* values (%) or medians (interquartile ranges). ^a^ is defined as the time elapsed between CPR and the ROSC. ^b^ indicates that CAG was performed before admission in all patients. ^c^ indicates that PCI was performed on a single vessel and before admission in all patients. ^d^ denotes *n* = 4. ^e^ denotes *n* = 5. ^f^ denotes *n* = 7. * denotes statistical significance at a 0.05 level compared with the low-severity group in a post hoc test after a Bonferroni correction. ** denotes statistical significance at a 0.01 level compared with the low-severity group in a post hoc test after a Bonferroni correction. *** denotes statistical significance at a 0.001 level compared with the low-severity group in a post hoc test after a Bonferroni correction. † denotes statistical significance at a 0.05 level compared with the moderate-severity group in a post hoc test after a Bonferroni correction. Abbreviations: COPD, chronic obstructive pulmonary disease; CPR, cardiopulmonary resuscitation; GCS, Glasgow Coma Scale; ROSC, return of spontaneous circulation; SOFA, sequential organ failure assessment; LAD, left anterior descending artery; LCx, left circumflex artery; RCA, right coronary artery; CAG, coronary angiography; VD, vessel disease; PCI, percutaneous coronary intervention.

**Table 3:** Multivariable linear regression identifying the factors independently associated with IL-6 level according to the injury severity or time.

Severity	NSE		CRP		PCT		CK-MB		TnI	
	β (95% CI)	*p*	β (95% CI)	*p*	β (95% CI)	*p*	β (95% CI)	*p*	β (95% CI)	*p*
Low	6.1 (−5.3; 17.5)	0.29	9.6 (−23.5; 42.6)	0.56	184.4 (−56.3; 425.1)	0.13	−1.5 (−3.7; 0.8)	0.21	−0.1 (−0.4; 0.1)	0.50
Moderate	4.3 ^a^ (0.1; 8.6)	0.04	1.2 (−47.4; 49.8)	0.96	17.0 ^a^ (5.9; 28.2)	0.003	1.2 (−3.6; 6.1)	0.62	−0.05 (−0.08; 0.02)	0.32
High	^b^ 7.9 (3.4; 12.5)	0.001	−24.8 (−92.9; 43.3)	0.47	−11.8 (−23.8; 0.2)	0.05	2.5 (−3.5; 8.5)	0.40	−0.1 (−0.2; 0.1)	0.99

^a^, adjusted R^2^ = 0.11. ^b^, adjusted R^2^ = 0.14. Abbreviations: NSE, neuron-specific enolase; CRP, C-reactive protein; PCT, procalcitonin; CK-MB, creatinine kinase-myocardial band; TnI, troponin I.

## Data Availability

The data presented here are available on request from the corresponding author. The data are not publicly available because of ethical concerns.
